# FROA-Drive: Failure-Routed Offline Adaptation for Lightweight Vision–Language–Action Autonomous Driving

**DOI:** 10.3390/s26185962

**Published:** 2026-09-20

**Authors:** Yunhan Xu, Ao Xu

**Affiliations:** 1 School of Automation, Nanjing University of Information Science and Technology, Nanjing 210044, China; 202413410028@nuist.edu.cn; 2 Shanghai Research Institute for Intelligent Autonomous Systems, Tongji University, Shanghai 200092, China

**Keywords:** autonomous driving, vision–language–action, offline adaptation, trajectory reranking, selective routing

## Abstract

Vision–Language–Action (VLA) models have shown strong potential for end-to-end autonomous driving, yet their post-training commonly relies on expensive simulator interaction or global policy updates. For an already competent pretrained policy, targeted correction is substantially more economical than repeated simulator interaction or global policy updates. We propose FROA-Drive, a lightweight offline adaptation framework that treats post-training as selective local correction. Starting from the frozen MindDrive-IL 0.5B checkpoint, FROA-Drive constructs a compact policy-centered candidate family around the original Top-1 speed trajectory, learns candidate-relative utility with a 1.51-million-parameter reranker, and uses a validation-calibrated gate to intervene only when an alternative is sufficiently supported. The original meta-actions and path trajectory are preserved by construction, providing an exact identity fallback and preventing unconstrained policy drift. On Chat-B2D-plus-10K, FROA-Drive achieves 72.8% candidate-selection accuracy and 84.2% intervention precision with a 5.4% proxy-violation rate. On Bench2Drive, it reaches a Driving Score of 77.08 and a Success Rate of 52.36%, improving the MindDrive-IL baseline by 1.23 DS and 3.06 percentage points in SR while requiring only 1.51 million trainable parameters and no online rollout during adaptation. These results establish selective local reranking as an effective, computation-efficient post-training strategy for lightweight driving VLA models.

## 1. Introduction

Autonomous driving requires a model to continuously perceive, reason, plan, and act in dynamic environments. The ultimate objective is therefore not isolated perception accuracy but robust closed-loop behavior under interaction, uncertainty, and distribution shift. Traditional modular pipelines separately optimize detection, tracking, prediction, and planning, which provides clear engineering interfaces but may also introduce information bottlenecks and error propagation. End-to-end autonomous driving (E2E-AD) has emerged as an alternative paradigm that directly learns planning-oriented representations from sensor observations [1]. Representative methods such as UniAD [2] and VAD [3] unify multiple driving tasks under planning-oriented or vectorized scene representations, while DriveTransformer [4] explores scalable Transformer-based driving architectures and DiffusionDrive [5] models multimodal actions with generative planning. Bench2Drive further shifts evaluation from open-loop trajectory similarity toward multi-ability closed-loop driving [6]. Together, these developments move the field from predicting a single expert-like trajectory toward learning policies that can repeatedly make reliable decisions in closed-loop environments.

Despite this progress, most E2E systems remain heavily dependent on expert demonstrations. Imitation learning is effective for acquiring basic driving skills, but it does not explicitly teach why one plausible action should be preferred over another, especially in rare or interactive situations. Vision–language models (VLMs) offer a new route by injecting semantic reasoning and world knowledge into driving. DriveLM organizes perception, prediction, and planning as graph-structured visual question answering [7]. SimLingo emphasizes explicit language–action alignment [8], while ORION connects vision–language reasoning to continuous trajectory generation [9]. OmniDrive further introduces counterfactual driving reasoning [10]. These efforts gradually transform autonomous driving from a vision-to-action problem into a vision–language–action (VLA) problem, where language is not only an explanation interface but also a high-level action abstraction.

Recent studies have therefore investigated reinforcement learning and preference-based post-training to go beyond imitation. MindDrive decomposes a lightweight VLA into a Decision Expert and an Action Expert, and it transfers exploration from the continuous trajectory space to a discrete linguistic meta-action space [11]. DriveDPO introduces preference optimization for safe driving trajectories [12], while Raw2Drive uses aligned world models to improve the scalability of reinforcement learning [13]. These approaches demonstrate the value of outcome-oriented post-training. Their computational profile also motivates a complementary direction: when a pretrained VLA already handles most states reliably, post-training can concentrate on the subset of predictions that benefit from local correction.

This observation motivates selective local adaptation: preserving the base policy while correcting a compact neighborhood around uncertain Top-1 predictions. Directly fine-tuning the entire model risks representation drift. Generating arbitrary alternatives from a large action space can simultaneously alter route semantics, lateral planning, and longitudinal motion. Conversely, always replacing the base output with a learned alternative may convert occasional ranking errors into systematic regressions. Efficient adaptation therefore requires both a constrained correction space and a principled criterion for deciding when to intervene.

To address these issues, we propose FROA-Drive (Failure-Routed Offline Adaptation for Lightweight Driving VLA). The core idea is *selective local correction*. We freeze the MindDrive-IL 0.5B checkpoint, including the visual encoder, language backbone, Decision Expert, and Action Expert. The original Top-1 prediction is always retained as candidate 0. Instead of sampling arbitrary new actions, FROA-Drive constructs a small policy-centered neighborhood by scaling only the forward component of the speed trajectory with a fixed set of factors, while preserving the speed meta-action, path meta-action, lateral coordinates, and complete path trajectory. This narrow candidate family isolates adaptation to longitudinal progress and avoids unconstrained changes to the semantic and lateral behavior of the base policy.

FROA-Drive then learns candidate quality entirely offline. Expert speed trajectories are used during training to compute the relative utility of each local candidate with respect to Top-1. A lightweight 1.51-million-parameter MLP reranker scores the candidates from frozen visual features, ego speed, base-policy actions, candidate geometry, and base confidence. The reranker is optimized with ranking, regression, and candidate-classification objectives, allowing it to learn ordering, improvement magnitude, and the discrete best candidate simultaneously. No additional large encoder, decoder, value network, or online rollout is required.

Finally, we introduce a validation-calibrated failure-routing gate. The reranker first proposes the best alternative and estimates its advantage over the original Top-1. The alternative is accepted only when this advantage exceeds a threshold calibrated on the validation set under non-regression constraints on speed quality and proxy risk. Otherwise, the system returns the original prediction exactly. The calibrated gate retains the frozen MindDrive output whenever the alternative does not satisfy the intervention criterion, yielding an exact identity fallback and evidence-based local correction.

Our contributions are summarized as follows:

- We propose a **policy-centered offline adaptation paradigm** for lightweight driving VLA models. FROA-Drive freezes the complete base policy and restricts adaptation to a compact longitudinal neighborhood around Top-1, avoiding costly online policy search and large-scale model updates.
- We introduce a **relative-utility candidate reranker** that uses offline expert supervision and a 1.51-million-parameter MLP with complementary ranking, regression, and classification objectives. The method requires neither a new large backbone nor simulator interaction during adaptation.
- We develop a **validation-calibrated selective routing mechanism with exact fallback**. FROA-Drive intervenes only when an alternative is sufficiently supported by the learned score, while preserving the original policy whenever the evidence is insufficient.

## 2. Related Work

### 2.1. End-to-End Autonomous Driving

Early E2E driving systems directly mapped visual observations to control commands or local trajectories. Conditional Imitation Learning incorporated high-level navigation commands to support route-dependent control [14], while Learning by Cheating transferred privileged scene knowledge to a vision-based policy [15]. These approaches establish the effectiveness of behavior cloning, but they also inherit the well-known distribution-shift problem of sequential imitation learning [16,17].

Subsequent research introduced structured intermediate representations and planning-oriented joint optimization. TransFuser fused camera and LiDAR features with Transformers [18], while TCP combined trajectory prediction with control prediction [19]. ThinkTwice [20] and DriveAdapter [21] improved scalable planning decoders and perception–planning coupling, respectively. UniAD [2] and VAD [3] further unified multiple driving tasks in end-to-end frameworks. These methods provide stronger inductive bias and better intermediate representations, yet they are still primarily optimized to reproduce expert behavior.

More recent work shifts from deterministic regression toward multimodal generation and world-model-based evaluation. GenAD formulates driving as generative future modeling [22], MomAD improves temporal consistency through momentum-aware planning [23], and BridgeAD incorporates historical prediction into current planning [24]. WoTE evaluates candidate trajectories by predicting their future BEV consequences [25], while World4Drive uses a physical latent world model for intention-aware planning [26]. Hydra-NeXt studies robust closed-loop driving from open-loop training [27], and CarPlanner combines generation and selection with reinforcement learning [28]. Bench2Drive [6] and NAVSIM [29] provide complementary closed-loop and data-driven evaluation protocols. These studies reveal a common trend: reliable driving increasingly depends on generating and evaluating alternatives rather than regressing a single trajectory. FROA-Drive follows this principle but replaces expensive online or world-model evaluation with a constrained offline candidate neighborhood around an already competent VLA policy.

### 2.2. Vision–Language Models and VLA for Driving

Large multimodal models have introduced semantic reasoning into autonomous driving. LMDrive demonstrates closed-loop driving with large language models [30], Feedback-Guided Autonomous Driving uses language feedback to improve policy learning [31], and DriveGPT4 connects video understanding, explanation, and control [32]. OpenEMMA explores open-source multimodal end-to-end driving [33], while SOLVE combines language–vision reasoning with conventional end-to-end networks [34]. DriveGPT4-V2 [35] and RoboTron-Drive [36] further extend closed-loop and unified multimodal driving capabilities.

A second line of work emphasizes language–action alignment and structured reasoning. DriveLM [7] organizes driving reasoning as graph-structured visual question answering, SimLingo [8] aligns language understanding with closed-loop driving, and OmniDrive [10] introduces counterfactual reasoning data for autonomous driving. VLR-Driver further studies large vision–language reasoning models for embodied driving [37]. ORION [9] connects high-level language reasoning to generative continuous trajectories, providing a direct bridge from semantic decisions to actions. Recent works such as DriveMoE [38], NoRD [39], ReCogDrive [40], OmniDrive-R1 [41], SpaceDrive [42], and MindDriver [43] continue to explore expert specialization, reasoning efficiency, reinforced cognition, and spatially grounded multimodal reasoning.

Recent VLA models increasingly treat language as an action abstraction rather than a pure explanation channel. AutoVLA combines adaptive reasoning with reinforcement fine-tuning [44], while OpenVLA demonstrates that large vision–language priors can be adapted to continuous control with parameter-efficient techniques [45]. Efficient adaptation is therefore a central requirement alongside stronger reasoning. Accordingly, FROA-Drive focuses on the downstream decision point where a frozen VLA already provides semantically valid trajectories and asks whether a small local alternative should replace the base output.

### 2.3. Reinforcement Learning and Efficient Post-Training

Reinforcement learning addresses the limitations of pure imitation by optimizing outcomes rather than expert similarity alone. PPO provides a stable policy-optimization framework [46], and CarPlanner applies reinforcement learning to trajectory generation and selection [28]. AlphaDrive introduces reinforcement learning for VLM-based autonomous driving reasoning [47], while VLM-RL directly combines vision–language models with safe driving reinforcement learning [48].

MindDrive [11] is particularly relevant because it moves online exploration from the continuous trajectory space into a discrete language meta-action space. A Decision Expert performs high-level reasoning, an Action Expert maps meta-actions to trajectories, and trajectory-level rewards are used to refine the decision policy. This design improves exploration efficiency but still relies on simulator-based rollout collection and policy optimization. Moreover, more rollout does not necessarily imply better performance, as MindDrive reports degradation when online updates become excessive.

Preference-based post-training provides another route. Direct Preference Optimization (DPO) removes the need for an explicit reward model in language-model alignment [49]. DriveDPO extends this idea to autonomous driving by constructing preferences from imitation similarity and safety scores [12]. IRL-VLA uses a reward world model to reduce the cost of computing driving rewards [50]. These methods demonstrate that relative preferences can provide useful post-training supervision. FROA-Drive differs in two respects: it does not update the large VLA itself, and it defines preferences over a deliberately narrow policy-centered candidate family with an explicit identity fallback.

### 2.4. Candidate Evaluation and Lightweight Adaptation

Candidate generation and evaluation are widely used to improve planning robustness. GenAD [22], CarPlanner [28], and WoTE [25] exploit multiple candidate futures or trajectories, while OmniDrive [10] shows that counterfactual alternatives are also useful in the language-reasoning space. However, evaluating a broad candidate set often requires a simulator, a world model, or a substantial learned planner.

For a pretrained VLA that already provides structured meta-actions and trajectories, a more economical strategy is to correct only a local neighborhood around Top-1. This perspective is compatible with parameter-efficient VLA adaptation [45] but moves the adaptation capacity even further downstream: the base policy remains frozen, and only a small reranker is learned. The remaining difficulty is selective intervention. Without a fallback, any ranking error can directly overwrite a correct base decision. FROA-Drive addresses this gap by combining policy-centered local candidates, offline relative-utility learning, and validation-calibrated routing, thereby turning global policy replacement into a lightweight and auditable selection problem.

## 3. Methods

### 3.1. Overview

**Figure 1:**
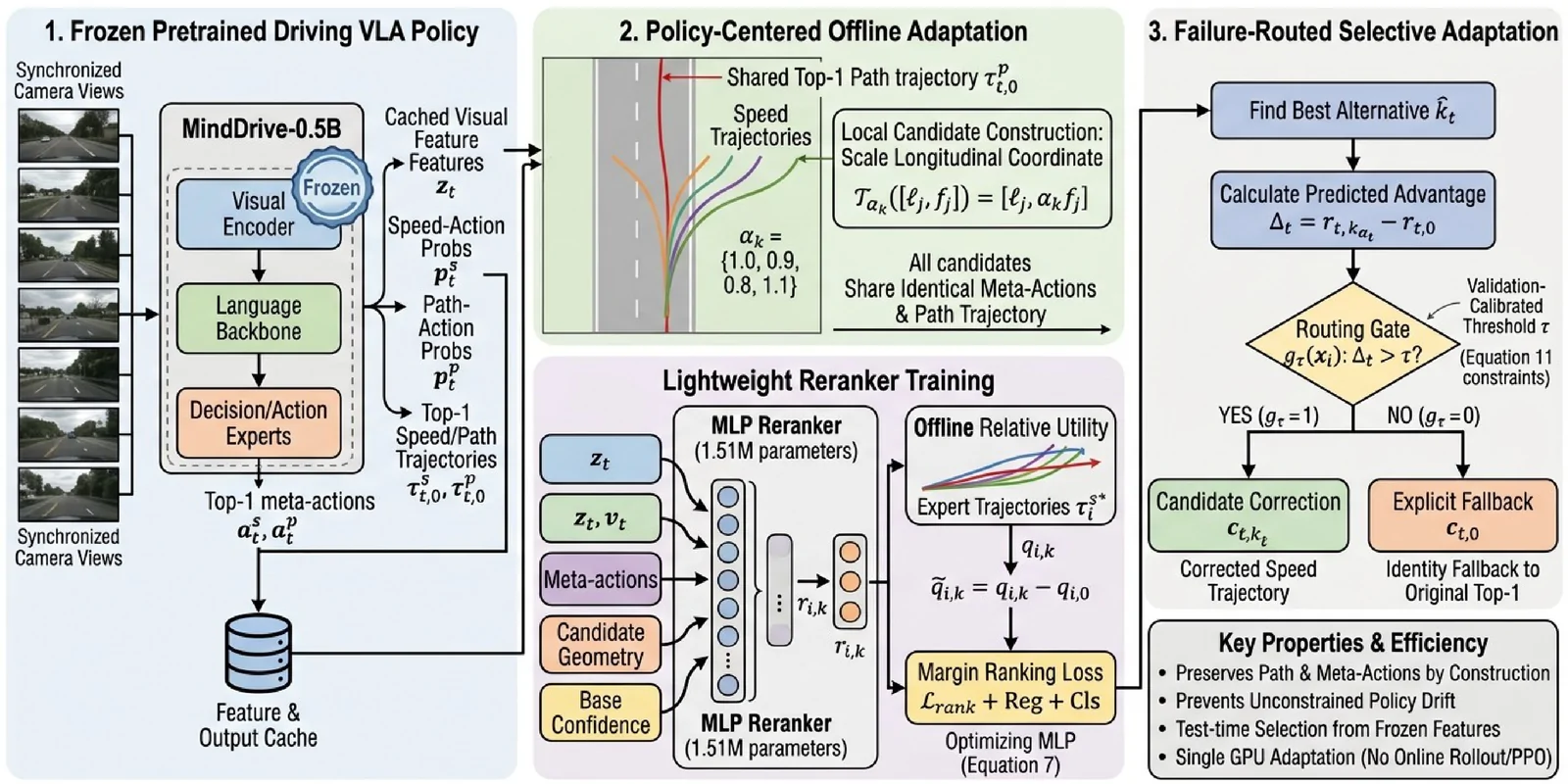
Overview of FROA-Drive. The frozen MindDrive-IL policy produces cached state features and Top-1 trajectories. A policy-centered candidate family is constructed around the original speed trajectory and scored by a lightweight reranker trained with offline relative utility. A validation-calibrated routing gate performs selective correction and preserves an exact identity fallback when the alternative is not sufficiently reliable.

Figure 1 illustrates the overall architecture of FROA-Drive. The framework contains three stages: First, the MindDrive-IL 0.5B policy is frozen and used to cache deployable visual features, action probabilities, and the original Top-1 speed/path trajectories. Second, a policy-centered local candidate family is built by applying small longitudinal scaling factors to the forward coordinates of the speed trajectory while preserving the original meta-actions and path trajectory. A lightweight MLP reranker is trained offline from relative utility with respect to expert speed trajectories. Third, a validation-calibrated routing gate compares the best alternative with the identity candidate and only accepts a correction when the predicted advantage is sufficiently reliable; otherwise, the original Top-1 output is returned exactly.

At time *t*, the driving state is

(1) $$x_{t}=\left\{I_{t}^{1:6},v_{t}\right\},$$

where $I_{t}^{1:6}$ denotes six synchronized camera views and $v_{t}$ is the ego speed. The frozen VLA policy $\pi_{0}$ predicts a speed meta-action $a_{t}^{s}$, a path meta-action $a_{t}^{p}$, a speed trajectory $\tau_{t}^{s}\in R^{T_{s}\times 2}$, and a path trajectory $\tau_{t}^{p}\in R^{T_{p}\times 2}$, with $T_{s}=6$ and $T_{p}=20$. Instead of updating $\pi_{0}$, we construct a candidate set $C_{t}={\left\{c_{t,k}\right\}}_{k=0}^{K-1}$, where candidate 0 is exactly the original Top-1 output. The adapted policy is

(2) $$\pi_{\mathrm{FROA}}\left(x_{t}\right)=\left\{\begin{array}{cc} c_{t,\hat{k}}, & g_{\tau }\left(x_{t}\right)=1,\\ c_{t,0}, & g_{\tau }\left(x_{t}\right)=0. \end{array}\right.$$

Thus, adaptation is formulated as selective candidate correction with an explicit fallback rather than global policy fine-tuning.

### 3.2. Policy-Centered Offline Adaptation

**Frozen VLA Features:** We retain the MindDrive-IL Qwen2-0.5B checkpoint, including its visual encoder, language backbone, Decision Expert, and Action Expert. A single frozen forward pass provides a visual feature $z_{t}\in R^{896}$, speed-action probabilities $p_{t}^{s}$, Top-1 speed/path action indices, and the corresponding speed/path trajectory banks. These outputs are cached in FP16 and reused throughout offline adaptation.

**Local Candidate Construction:** Arbitrary action sampling can simultaneously alter semantic decisions, lateral planning, and longitudinal behavior. We instead construct a policy-centered longitudinal neighborhood around the base prediction. Let $\tau_{t,0}^{s}$ and $\tau_{t,0}^{p}$ denote the Top-1 speed and path trajectories. For a scale $\alpha_{k}$, we transform only the forward coordinate:

(3) $$T_{\alpha_{k}}\left(\left[\ell_{j},f_{j}\right]\right)=\left[\ell_{j},\alpha_{k}f_{j}\right].$$

We use A={1.0,0.9,0.8,1.1} and K=4. The resulting candidate is

(4) $$c_{t,k}=\left({\hat{a}}_{t}^{s},{\hat{a}}_{t}^{p},T_{\alpha_{k}}\left(\tau_{t,0}^{s}\right),\tau_{t,0}^{p},p_{t}^{s}\left({\hat{a}}_{t}^{s}\right)\right).$$

**Figure 2:**
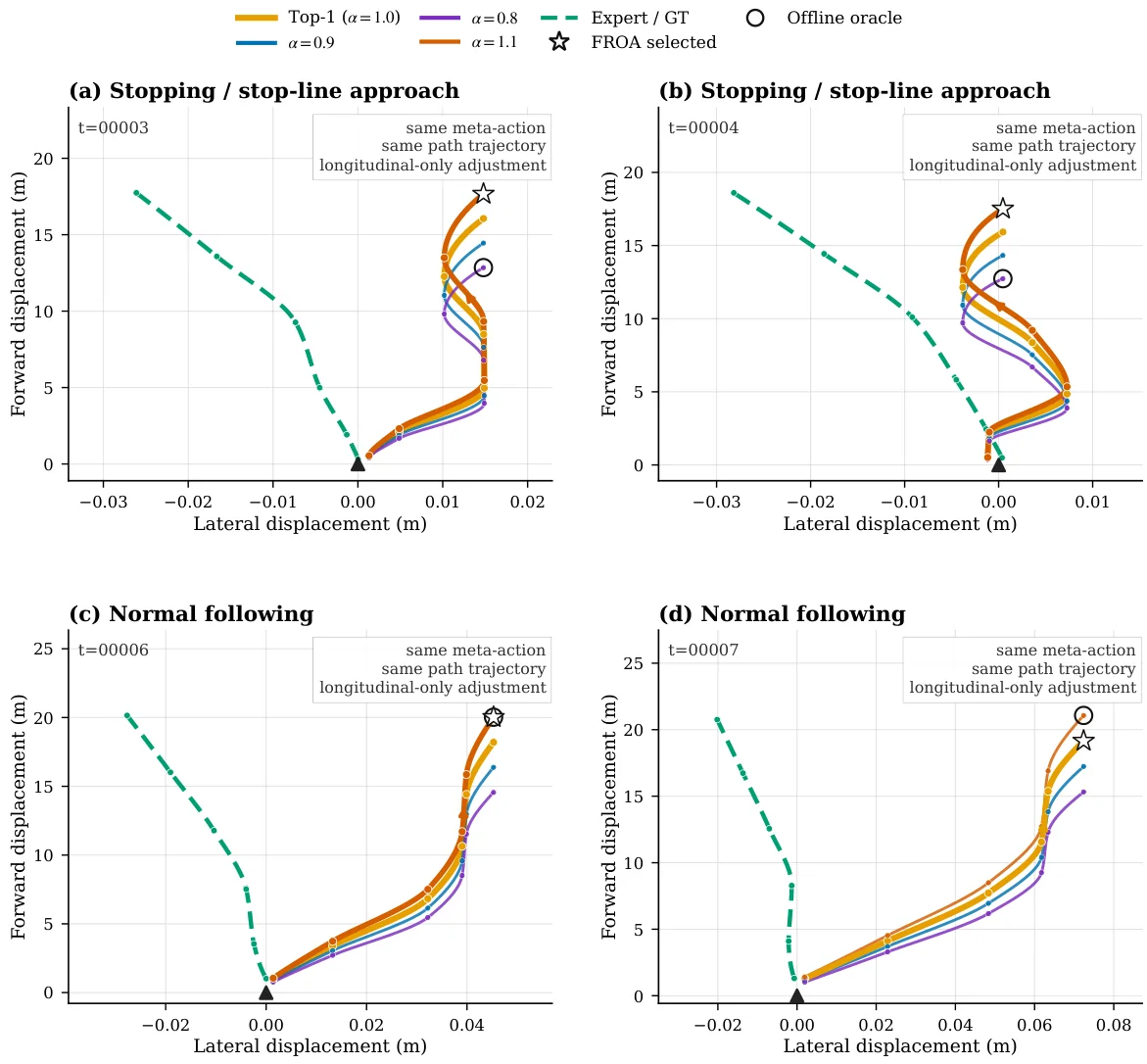
Policy-centered candidate-family visualization. Each panel shows the original Top-1 trajectory (α=1.0) and three longitudinal alternatives (α∈{0.9,0.8,1.1}) that share the same base meta-actions and path trajectory. The star denotes the candidate selected by FROA-Drive, the circle denotes the offline oracle, and the expert/GT trajectory is shown only for post hoc analysis. The visualization makes explicit that adaptation is restricted to longitudinal correction rather than unconstrained replanning.

All candidates share the same meta-actions and full path trajectory. Candidate 0 (α=1) is the unmodified base output, while the remaining candidates provide conservative or slightly more progressive longitudinal alternatives. Figure 2 visualizes this bounded candidate family on representative test frames. The expert trajectory and offline-oracle marker are shown only for analysis; neither is used for candidate construction or test-time routing.

**Offline Relative Utility:** During training, expert trajectories are used only to construct supervision. For candidate *k*, the speed imitation error is

(5) $$L_{\mathrm{spd}}\left(i,k\right)=\frac{1}{2T_{s}}{∥\tau_{i,k}^{s}-\tau_{i}^{s*}∥}_{1}.$$

Our implementation uses

(6) $$q_{i,k}=-L_{\mathrm{spd}}\left(i,k\right),\;{\tilde{q}}_{i,k}=q_{i,k}-q_{i,0},$$

so the learner directly estimates whether an alternative improves upon Top-1. Safety, rule, and comfort diagnostics are retained for validation-time constraints rather than being mixed into the primary learning target.

**Lightweight Reranker:** Each candidate is represented by the frozen visual feature, ego speed, base meta-actions, candidate speed/path geometry, and base confidence. The resulting 963-dimensional feature vector is scored by a shared MLP with hidden dimensions 1024 and 512, containing 1,512,449 trainable parameters. Let $k_{i}^{+}$ and $k_{i}^{-}$ denote the best and worst candidates according to $\tilde{q}$. We optimize

(7) $$L=L_{\mathrm{rank}}+\lambda_{\mathrm{reg}}L_{\mathrm{reg}}+\lambda_{\mathrm{cls}}L_{\mathrm{cls}},$$

where $L_{\mathrm{rank}}$ is a pairwise margin loss, $L_{\mathrm{reg}}$ regresses normalized relative utility, and $L_{\mathrm{cls}}$ classifies the best candidate. We use margin m=0.25, $\lambda_{\mathrm{reg}}=0.1$, and $\lambda_{\mathrm{cls}}=1.0$.

### 3.3. Failure-Routed Selective Adaptation

Always accepting the reranker’s output can turn small ranking errors into systematic policy regressions. FROA-Drive therefore treats the score gap over Top-1 as evidence that the base policy may require correction. The best alternative is

(8) $${\hat{k}}_{i}=\arg\max_{k\in \{1,\ldots ,K-1\}}r_{i,k},$$

with predicted advantage

(9) $$\Delta_{i}=r_{i,{\hat{k}}_{i}}-r_{i,0}.$$

We intervene only when

(10) $$g_{\tau }\left(x_{i}\right)=⊮\left[\Delta_{i}>\tau \right].$$

The threshold τ is calibrated exclusively on the validation split. Among all candidate thresholds, we choose the one with maximum routing coverage subject to

(11) $$M_{\mathrm{spd}}\left(\tau \right)\leq M_{\mathrm{spd}}^{\mathrm{Top}1},\;M_{\mathrm{risk}}\left(\tau \right)\leq M_{\mathrm{risk}}^{\mathrm{Top}1},$$

where $M_{\mathrm{spd}}$ and $M_{\mathrm{risk}}$ denote validation speed error and proxy-risk rate. If no alternative threshold satisfies the constraints, routing is disabled and the model exactly recovers MindDrive Top-1.

This design has three useful properties: First, path and meta-action outputs are invariant across candidates. Second, the original prediction is always available as an exact fallback. Third, test-time selection uses only deployable frozen features and candidate geometry; no expert labels, simulator, online rollout, PPO objective, or additional Transformer is required. The adaptation cost is dominated by one-time feature caching and training a 1.51-million-parameter MLP, making the method suitable for a single RTX 4090.

## 4. Experiments

### 4.1. Experimental Settings

**Dataset:** We used Bench2Drive Base together with the frame-level language and meta-action annotations from Chat-B2D-plus. To keep the storage and training budget practical for a single GPU, we constructed **Chat-B2D-plus-10K**, a clip-disjoint subset containing 10,240 annotated frames from 120 clips and occupying 46.3 GB. The subset was divided into train/validation/test splits at an 8:1:1 ratio at the clip level to avoid leakage between adjacent frames. All internally evaluated results reported in this study are arithmetic means over five independent runs with different random seeds under the same clip-disjoint split and evaluation protocol. Published comparison rows retain the values reported by their original papers. Small numerical differences are interpreted as average performance trends rather than formal statistical-significance claims.

**Model and Training:** The frozen backbone was the MindDrive-IL Qwen2-0.5B checkpoint [11], i.e., the model after imitation learning and before online RL. All VLA parameters remained fixed. We cached the 896-dimensional visual feature, action probabilities, Top-1 action indices, and trajectory banks once for each frame. The candidate set used A={1.0,0.9,0.8,1.1}. The reranker contained 1.51 million trainable parameters and was optimized with AdamW using a learning rate of $3\times 10^{-4}$, weight decay $10^{-4}$, FP16, and validation-based early stopping. All adaptation experiments were performed on a single NVIDIA RTX 4090 24 GB GPU. Here, offline adaptation refers to the training and calibration protocol rather than to the evaluation environment. The reranker and routing threshold were learned exclusively from offline data. After training and calibration were complete, all parameters and the threshold were frozen, and the resulting policy was evaluated in the Bench2Drive closed-loop simulator only for measurement; simulator trajectories were not fed back into training, threshold selection, or parameter updates.

**Metrics:** Because the candidate family preserves the path trajectory and meta-actions by construction, we focus on longitudinal adaptation and selective routing. We report Candidate Selection Accuracy (CSA), Speed-L1, oracle gap (OG), Proxy Violation Rate (PVR), routing coverage (Cov.), intervention precision (IP), and the Bench2Drive closed-loop Driving Score (DS) and Success Rate (SR). In this paper, “proxy risk” denotes the offline rule-violation diagnostic attached to each candidate, rather than a learned collision probability. PVR is the percentage of evaluated frames for which the finally selected candidate has a nonzero rule-violation count produced by the offline candidate scorer. Routing coverage is the percentage of frames on which the gate accepts an alternative, while intervention precision is the percentage of routed frames for which the accepted alternative has higher expert-referenced relative utility than the original Top-1 candidate. CSA and OG evaluate candidate ranking, whereas coverage, intervention precision, PVR, and closed-loop DS/SR evaluate the routing mechanism and its downstream effect. We additionally report trainable parameters, peak memory, feature-caching cost, adaptation time, and added inference latency. For the qualitative cases in Figure 4, ADE and FDE are reported as auxiliary geometric trajectory errors.

### 4.2. Main Results

**Open-Loop Candidate Selection:** Table 1 evaluates candidate-selection strategies on Chat-B2D-plus-10K. All learned methods share the same frozen backbone and the same K=4 local candidate family. Always-rerank yields the lowest Speed-L1 because it modifies every sample, while the complete FROA-Drive reduces PVR to 5.4% and improves intervention precision to 84.2% with a routing coverage of 31.8%. This demonstrates the role of selective correction: the calibrated gate increases intervention precision and lowers PVR while retaining strong open-loop accuracy.

**Closed-Loop Bench2Drive Comparison:** Table 2 compares FROA-Drive with representative E2E and VLA methods. MindDrive-IL is the direct baseline because it is the frozen initialization used by FROA-Drive. Offline adaptation raises its performance from 75.85 to 77.08 DS and from 49.30% to 52.36% SR, corresponding to gains of 1.23 DS and 3.06 percentage points in SR. MindDrive-RL reaches 78.04 DS and 55.09% SR after online reinforcement learning and is reported as a separate post-training reference. We additionally include DriveDPO as a published offline preference-optimization reference on Bench2Drive. Its sensor configuration and backbone differ from the MindDrive-based setting, so the row is used for benchmark context rather than as a same-backbone controlled ablation. The FROA-Drive row is the mean of five independent runs, while values for published competitors are reproduced from their corresponding papers.

**Efficiency:** Table 3 reports adaptation efficiency. The large-model forward pass is only required during one-time feature caching. The actual trainable component contains 1.51 million parameters and requires 0.42 h for adaptation after caching, with 0.7 ms of additional inference latency. This separation makes repeated ablation and threshold studies substantially cheaper than full-model or simulator-based post-training.

### 4.3. Ablation Studies

**Component Analysis:** Table 4 isolates the contributions of relative utility, the multi-objective reranking loss, and calibrated routing. Relative utility and the combined ranking objective progressively reduce Speed-L1 and improve CSA. Adding the calibrated gate lowers PVR from 6.0% to 5.4% and produces the best closed-loop result (77.08 DS and 52.36% SR), while Speed-L1 remains 0.151. The gain verifies the gate as a reliability-oriented routing mechanism.

**Candidate-Family Design:** Table 5 studies the local trust region. Increasing *K* expands the correction range but also makes candidate selection harder. The four-candidate family {1.0,0.9,0.8,1.1} achieves the best closed-loop DS while retaining low Speed-L1 and PVR, indicating a favorable balance between correction coverage and distribution shift.

**Figure 3:**
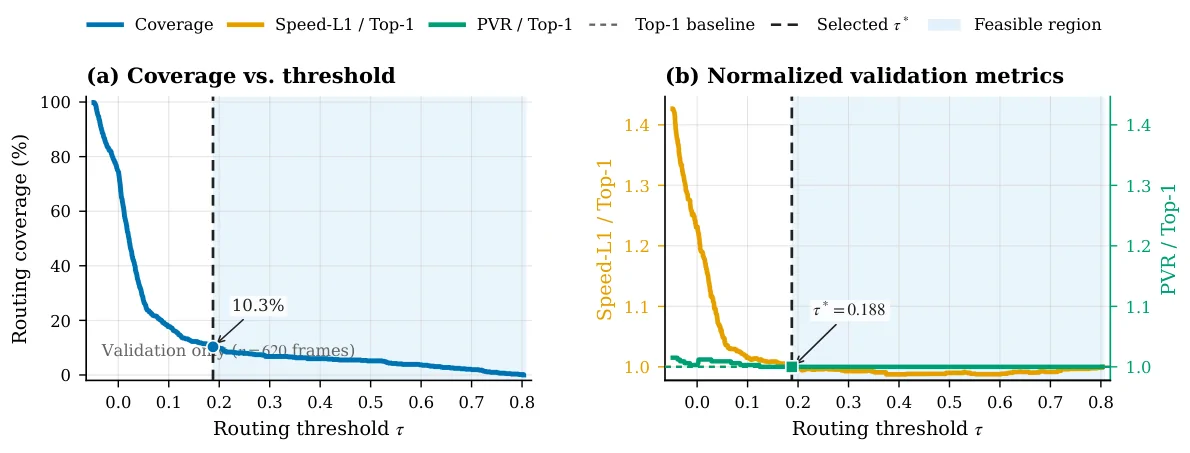
Validation calibration of the failure-routing threshold. (**a**) Routing coverage decreases with the score-gap threshold; the selected $\tau^{*}=0.188$ gives 10.3% validation coverage. (**b**) Speed-L1 and PVR are normalized by their respective Top-1 values, so 1.0 is the non-regression reference. The dashed vertical line marks $\tau^{*}$, and the shaded region denotes thresholds satisfying Equation (11). Calibration uses 620 validation frames only.

**Routing Calibration:** Table 6 studies the coverage–reliability trade-off. Always reranking provides the lowest Speed-L1, whereas non-regression calibration achieves the best closed-loop result, with 31.8% test routing coverage, 84.2% intervention precision, and 5.4% PVR. Figure 3 shows the validation sweep used to select $\tau^{*}=0.188$ from 620 calibration frames. At the selected threshold, validation coverage is 10.3%; held-out test statistics are reported independently in Table 6. The 10.3% coverage in Figure 3 is the validation operating-point coverage used to choose the fixed threshold, whereas the 31.8% coverage in Table 1 and Table 6 is obtained after applying that fixed threshold to the held-out test split. These values correspond to different data partitions. Selective routing therefore improves closed-loop reliability over unconditional reranking.

### 4.4. Qualitative Analysis

**Figure 4:**
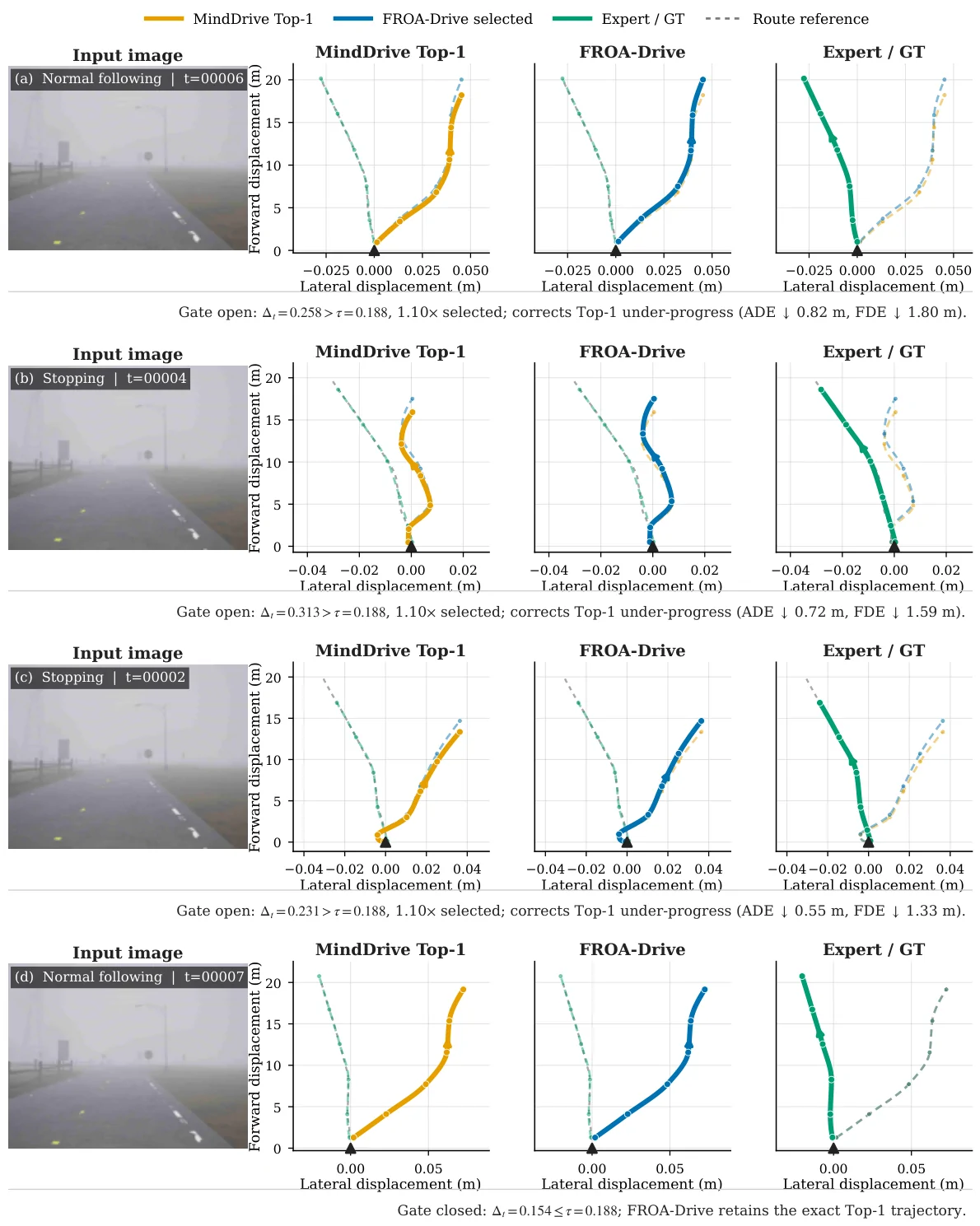
Qualitative comparison in representative driving scenarios. Orange, blue, and green denote the MindDrive Top-1, the FROA-Drive output, and expert/GT trajectories, respectively; the dashed curve is the route reference. Rows (**a**–**c**) are routed examples with $\Delta_{t}>\tau$ in which the 1.10× local candidate corrects Top-1 under-progress and reduces ADE/FDE. Row (**d**) is a non-routed example with $\Delta_{t}\leq \tau$, demonstrating exact fallback to the original Top-1. Expert/GT is displayed only for analysis and is not available to the test-time gate.

Figure 4 visualizes routed and non-routed cases from the clean offline test subset. Rows (a)–(c) accept the 1.10× alternative when $\Delta_{t}>\tau$, reducing ADE/FDE relative to Top-1, whereas row (d) keeps the gate closed and exactly preserves the original trajectory. The examples directly connect the reranker’s local proposal to the selective routing behavior quantified in Table 6.

## 5. Discussion and Limitations

The term “failure-routed” in this work refers to selective correction inside the bounded longitudinal candidate family, rather than to universal correction of every driving failure. The current candidates preserve the base speed/path meta-actions, lateral coordinates, and complete path trajectory. This restriction provides a controlled trust region and exact fallback, but it also means that FROA-Drive cannot repair an incorrect lane choice, lateral path, route semantic decision, or other failure that requires changing the frozen path or meta-actions. The qualitative cases therefore focus on longitudinal progress errors such as stopping and following. Extending the candidate family to lateral and semantic alternatives while retaining explicit fallback is an important direction for future work.

Chat-B2D-plus-10K contains 10,240 frames, so overfitting of the reranker remains a relevant concern. The current protocol limits this risk through separate clip-disjoint training, validation, and test partitions, a frozen VLA backbone, a 1.51-million-parameter downstream reranker, validation-based early stopping, and five independent random-seed runs for internally evaluated results. The improvement is observed on the held-out open-loop split and in the separate Bench2Drive closed-loop evaluation. Broader cross-dataset validation and larger offline training sets would provide stronger evidence of generalization and are left for future study.

The routing gate is evaluated in the present study through coverage, intervention precision, PVR, and downstream closed-loop DS/SR, while CSA and oracle gap characterize the underlying candidate ranking. Recall, AUROC, and AUPRC would provide an additional threshold-independent view if routing were cast as a binary beneficial-intervention classification problem. A future evaluation will report these discrimination metrics together with same-backbone PEFT and DPO baselines trained under an identical MindDrive-IL initialization and Bench2Drive protocol. Table 2 includes the published DriveDPO result for contextual comparison, and Table 3 includes Decision-LoRA preference fine-tuning as a computational-efficiency reference.

## 6. Conclusions

We present FROA-Drive, a lightweight offline adaptation framework that reformulates driving-VLA post-training as selective local correction. Starting from the MindDrive-IL 0.5B checkpoint, FROA-Drive constructs a compact longitudinal candidate neighborhood around the Top-1 trajectory and learns candidate-relative utility with a 1.51-million-parameter reranker. A validation-calibrated gate selects reliable corrections and preserves the original output through exact identity fallback. On Chat-B2D-plus-10K, the proposed routing strategy reaches 84.2% intervention precision with a 5.4% proxy-violation rate. On Bench2Drive, FROA-Drive improves its frozen MindDrive-IL initialization from 75.85 to 77.08 DS and from 49.30% to 52.36% SR. The complete adaptation requires 0.42 h of reranker training after one-time feature caching on a single RTX 4090 and introduces 0.7 ms additional inference latency. These results demonstrate that policy-centered candidate reranking and selective routing provide an effective offline post-training path for lightweight driving VLA models. The present formulation is limited to longitudinal correction and a 10K-frame offline adaptation set; future work will extend the candidate space to lateral and semantic alternatives, broaden cross-dataset validation, and evaluate threshold-independent routing metrics and same-backbone PEFT/DPO comparisons under a unified closed-loop protocol.

## Figures and Tables

**Table 1:** Open-loop local candidate selection on the held-out test split of Chat-B2D-plus-10K.

Method	CSA (%) ↑	Speed-L1 ↓	OG ↓	PVR (%) ↓	Cov. (%)	IP (%) ↑
MindDrive Top-1 [11]	58.7	0.184	0.037	7.8	0.0	–
Fixed α=0.9	22.6	0.172	0.025	6.9	100.0	61.4
Rule-Gated Local Correction	63.8	0.164	0.017	6.2	27.5	74.1
Regression-only Reranker	69.5	0.158	0.011	6.4	100.0	70.8
Ranking-only Reranker	71.2	0.154	0.009	6.2	100.0	72.3
FROA-Drive without Gate	**73.4**	**0.145**	**0.004**	6.0	100.0	74.6
**FROA-Drive**	72.8	0.151	0.006	**5.4**	31.8	**84.2**
Local Oracle	100.0	0.141	0.000	4.8	–	–

**Table 2:** Closed-loop comparison on Bench2Drive. MindDrive-IL is the frozen base policy; MindDrive-RL is the online post-training reference.

Method	Paradigm	Backbone/LLM	Training	DS ↑	SR (%) ↑
TCP-traj [19]	E2E	CNN	IL	59.90	30.00
ThinkTwice [20]	E2E	CNN/Transformer	IL	62.44	31.23
UniAD [2]	E2E	Transformer	IL	45.81	16.36
VAD [3]	E2E	Transformer	IL	42.35	15.00
DriveTransformer [4]	E2E	Transformer	IL	63.46	35.01
Raw2Drive [13]	E2E	World Model	Model-based RL	71.36	50.24
DriveDPO [12]	E2E	TransFuser/ResNet-34	Offline DPO	62.02	30.62
ORION-Qwen2-0.5B [9]	VLA	Qwen2-0.5B	IL	72.89	45.83
MindDrive-IL (base) [11]	VLA	Qwen2-0.5B	IL	75.85	49.30
MindDrive-RL [11]	VLA	Qwen2-0.5B	Online RL	**78.04**	**55.09**
**FROA-Drive**	VLA	Qwen2-0.5B	Offline adaptation	77.08	52.36

**Table 3:** Adaptation efficiency on a single RTX 4090.

Method	Trainable Params	Online Rollout	Peak VRAM	One-Time Cache	Adapt. Train	Extra Latency
Fixed Local Correction	0	No	18.4 GB	3.1 h	0 h	<0.1 ms
Rule-Gated Local Correction	0	No	18.4 GB	3.1 h	<0.1 h	0.1 ms
Decision-LoRA Preference FT	8.2 M	No	22.1 GB	3.1 h	2.8 h	∼0 ms
FROA-Drive without Gate	1.51 M	No	18.4 GB	3.1 h	0.42 h	0.6 ms
**FROA-Drive**	**1.51 M**	**No**	**18.4 GB**	**3.1 h**	**0.42 h**	**0.7 ms**
MindDrive-RL [11]	LoRA + Value	Yes	Multi-GPU	–	Multi-simulator	–

**Table 4:** Component-wise ablation.

Relative Utility	Rank + Reg + Cls	Calibrated Gate	CSA (%) ↑	Speed-L1 ↓	PVR (%) ↓	DS ↑	SR (%) ↑
–	–	–	58.7	0.184	7.8	75.85	49.30
			66.9	0.162	6.7	76.21	49.94
🗸			69.8	0.155	6.4	76.43	50.61
🗸	🗸		**73.4**	**0.145**	6.0	76.72	51.28
🗸	🗸	🗸	72.8	0.151	**5.4**	**77.08**	**52.36**

**Table 5:** Ablation on the policy-centered candidate family.

A	*K*	CSA	Speed-L1	PVR	DS
{1.0,0.9}	2	76.1	0.160	5.9	76.58
{1.0,0.9,0.8}	3	74.6	0.154	5.6	76.86
{1.0,0.9,0.8,1.1}	4	72.8	**0.151**	**5.4**	**77.08**
{1.0,0.9,0.8,0.7,1.1,1.2}	6	68.3	0.149	6.1	76.64

**Table 6:** Ablation on selective routing.

Routing Strategy	Cov. (%) ↓	IP (%) ↑	Speed-L1 ↓	PVR (%) ↓	DS ↑	SR (%) ↑
Top-1/No Intervention	0.0	–	0.184	7.8	75.85	49.30
Always Rerank	100.0	74.6	**0.145**	6.0	76.72	51.28
Score Gap τ=0	56.7	78.9	0.147	5.8	76.83	51.71
Val. Speed-Only Calibration	43.2	81.1	0.146	6.3	76.61	51.44
**Non-Regression Calibration**	**31.8**	**84.2**	0.151	**5.4**	**77.08**	**52.36**

## Data Availability

The original data presented in the study are openly available in Bench2Drive at https://github.com/Thinklab-SJTU/Bench2Drive (accessed on 18 September 2026).
